# Use of Multimodal Mechanical Support in Refractory Cardiogenic Shock From Bupropion Overdose

**DOI:** 10.7759/cureus.45126

**Published:** 2023-09-12

**Authors:** Kevin Bodker, Abdulghani Mounir, Nathan Marzlin, Mirza Baig, Jayant Khitha

**Affiliations:** 1 Cardiology, Medical University of South Carolina, Charleston, USA; 2 Aurora Cardiovascular and Thoracic Services, Aurora Sinai/Aurora St. Luke's Medical Centers, Milwaukee, USA

**Keywords:** vt storm, mars, impella, ecmo, bupropion overdose

## Abstract

Massive bupropion overdose has a known association with cardiogenic shock. We describe a clinical case of a 48-year-old female who was brought to the hospital by emergency medical services after ingesting numerous psychiatric medications. Her hospital course was complicated by worsening cardiogenic shock and ventricular tachycardia storm. Transthoracic echocardiography showed left ventricular (LV) hypokinesis with an ejection fraction of 9%. Then the patient underwent placement of a percutaneous Impella CP device (Abiomed, Danvers, MA). The Molecular Adsorbent Recirculating System was started for protein-bound bupropion clearance. After 24 hours, the patient returned to an organized sinus rhythm. A repeat echocardiogram done on the next day demonstrated improved LV function, and the patient had profound clinical improvement. The case illustrates how the use of extracorporeal membrane oxygenation in combination with Impella device and Molecular Adsorbent Recirculating System was able to support the patient's recovery.

## Introduction

Massive bupropion overdose has a known association with cardiogenic shock (CS). We describe a case of CS refractory to venoarterial (VA) extracorporeal membrane oxygenation (ECMO). The case illustrates how the use of ECMO in combination with an Impella ventricular assistance device (Abiomed, Danvers, MA) and Molecular Adsorbent Recirculating System (Teraklin AG, Rostock, Germany) was able to support the patient's recovery.

## Case presentation

**Figure 1:**
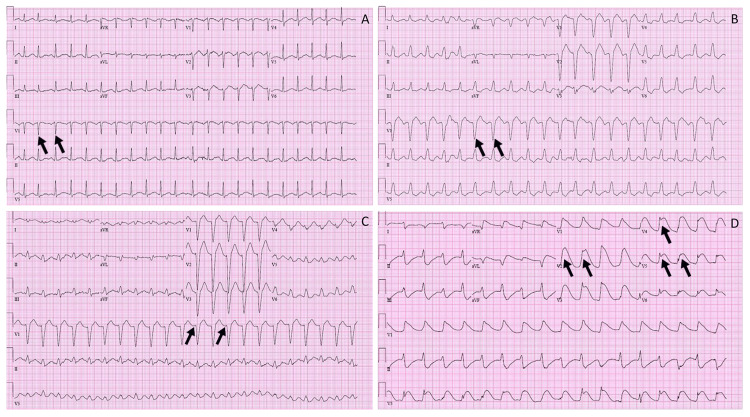
Progression of electrocardiograms throughout the initial decompensation Panel A shows the presenting electrocardiogram showing atrial fibrillation with a rapid ventricular response of 136 with QTc of 575 msec and QRS duration of 90 msec. Panel B shows a wide complex tachycardia (121 bpm) with a left bundle branch block that has replaced atrial fibrillation with QTc of 545 msec. Panel C shows a similar rhythm with prolongation of QTc to 583 bpm along with a shortened PR interval, and Panel D shows wide complex tachycardia with diffuse ST abnormalities.

A 48-year-old female was brought to the hospital by emergency medical services after ingesting numerous psychiatric medications. It was unclear when the patient took the medications in relation to the time emergency responders arrived. Multiple empty pill bottles, including escitalopram 20 mg, bupropion XL 150 mg, lorazepam 0.5 mg, and mirtazapine 15 mg, were found. Physical examination at the emergency department was significant for sinus tachycardia and altered mental status with a Glasgow coma scale score of 11. Electrocardiograms obtained throughout the initial decompensation are shown in Figure 1. The presenting electrocardiogram (Figure 1A) showed atrial fibrillation with a rapid ventricular response of 136 with QTc of 575 msec and QRS duration of 90 msec. Shortly after arrival, the patient had a 30-second tonic-clonic seizure, which resolved spontaneously with lorazepam. The patient was subsequently intubated in the emergency department owing to her declining mental status with concerns for airway compromise. Poison Control recommended magnesium sulfate 2 g intravenous (IV) and three doses of sodium bicarbonate (100 mEq/50 mL). She was admitted to the intensive care unit (ICU) for further management.

Initial arterial blood gas was unremarkable. The metabolic panel was without any electrolyte derangements or signs of kidney or liver dysfunction, and the complete blood count was normal. Alcohol and toxicology screenings were negative. She developed leukocytosis within 12 hours of admission with a white blood cell count of 21 K/mcl. Troponin was slightly elevated at 0.47 (ng/mL), and the electrocardiogram showed ST elevations that were not deemed ischemic owing to the clinical picture and lack of risk factors. A bedside point-of-care heart ultrasound was performed and showed grossly normal left and right ventricular function. 

Shortly after admission to the ICU, the patient became hypotensive, initially requiring norepinephrine infusion to maintain blood pressure. Within a few hours, the patient continued to decompensate with increasing pressor requirements. Before being transferred to a tertiary care facility, the patient was dosed with intravenous lipid solution as escitalopram and bupropion are lipid soluble. The patient was started on a sodium bicarbonate drip on arrival. 

Twenty hours into her hospitalization, telemetry showed polymorphic ventricular tachycardia. Cardiopulmonary resuscitation was initiated, and external defibrillation was utilized due to ventricular tachycardia (VT). Return of spontaneous circulation was achieved within two minutes. She was noted to have a QTc of >800 at that time. Electrophysiology was consulted and recommended multiple repeat doses of magnesium and started lidocaine and esmolol infusions for VT storm. The patient remained predominately in monomorphic VT refractory to lidocaine infusion (Figure 1D). After evaluation by cardiothoracic surgery, the patient was placed on VA-ECMO for CS and continued VT storm. Despite VA-ECMO and maximal medical support, the patient developed a sine wave on telemetry. Transthoracic echocardiography showed profound global left ventricular (LV) hypokinesis with an ejection fraction of 9%. After discussion with the care team, the patient underwent placement of a percutaneous Impella CP device. The Molecular Adsorbent Recirculating System was started for protein-bound bupropion clearance. After 24 hours, the patient returned to an organized sinus rhythm. A repeat echocardiogram the next day demonstrated improved LV function with an ejection fraction of 25%. The Impella device was removed, and ECMO was weaned the same day. Molecular Adsorbent Recirculating System therapy was continued for 48 hours. The patient maintained a sinus rhythm and was extubated 48 hours after ECMO decannulation. Four days later, a limited echocardiogram showed normal LV function and an ejection fraction of 53%. The patient was eventually discharged to an inpatient psychiatric facility for further treatment.

## Discussion

There are a handful of available case reports on massive bupropion overdoses with a wide range of therapeutic attempts, some apparently successful and others less so. Assessing the efficacy of particular supportive care is not possible given the small sample size, but we reviewed the literature attempting to draw correlations between interventions and outcomes. Cardiotoxicity with varying degrees of CS appears common in case reports, with most identifying early and severely impaired systolic function that required pressor support. Conduction abnormalities are invariably reported as conduction delays with prolonged QRS and correct QT intervals. After ingestion of large amounts of bupropion, most cases presented with sinus tachycardia with further rhythm abnormalities developing in the hours to one day after arrival. These abnormalities include frequent premature ventricular complexes [1], nodal rhythm with premature junctional complexes [2], and wide complex tachycardias, as seen in our case and others [3]. Delayed sinus bradycardia also has been described [4]. Sodium bicarbonate infusion was used in most cases with unclear efficacy. Lipid emulsion therapy, in which intravenous lipids are administered for overdoses of lipophilic drugs, such as bupropion, was frequently utilized. In the case of CS with severe LV dysfunction, only a single case of the use of an LV assist device could be found. Reaume et al. [5] described their use of an Impella device for bupropion-induced CS along with other supportive care. The cardiotoxicity of bupropion overdose proved fatal 36 hours following admission despite the use of Impella. The authors recommended consideration of ECMO for similar clinical cases in the future. VA-ECMO was implemented in a case series of two patients with eventual recovery of both patients [6]. As it applies to the case presented here, the use of mechanical circulatory support is supported in severe CS and persistently unstable arrhythmias. LV assist devices, such as Impella, are thought to be more efficacious in profound CS, given their continuous nature of support and degree of increased cardiac output. If available, the use of ECMO is supported if the aforementioned interventions fail to improve oxygenation adequately. To our knowledge, there is no literature that shows MARS being used with ECMO and Impella in refractory cardiogenic shock secondary to toxic ingestion of bupropion.

## Conclusions

Massive bupropion overdose has a known association with cardiogenic shock and can lead to poor outcomes. This clinical case highlights the complications patients can have, such as severe cardiogenic shock and refractory ventricular tachycardia. Molecular adsorbent recirculating therapy was not enough to support the patient's recovery, and she ultimately required advanced cardiac supportive devices. The goal of this case is to illustrate how the use of VA-ECMO in combination with Impella and Molecular Adsorbent Recirculating System was able to support the patient's recovery.
